# Moderation Effect of Handgrip Strength on Cognition and Functional Independence Associations in Adults Over 90 Years

**DOI:** 10.1002/jcsm.13838

**Published:** 2025-06-04

**Authors:** Mikel L. Sáez de Asteasu, Eduardo L. Cadore, Tainara Steffens, Eduarda Blanco‐Rambo, Talita Molinari, Marcelo Bandeira‐Guimaraes, Mikel Izquierdo, Caroline Pietta‐Dias

**Affiliations:** ^1^ Navarrabiomed, Hospital Universitario de Navarra (HUN) Universidad Pública de Navarra (UPNA), IdiSNA Pamplona Spain; ^2^ CIBER of Frailty and Healthy Aging (CIBERFES) Instituto de Salud Carlos III Madrid Spain; ^3^ Exercise Research Laboratory, School of Physical Education, Physiotherapy and Dance Universidade Federal do Rio Grande do Sul Porto Alegre Brazil

**Keywords:** Centenarians, Cognition, handgrip, Strength

## Abstract

**Introduction:**

The causal relationship between cognitive impairment and functional decline seems bidirectional in older adults. This study aimed to determine the moderating effect of handgrip strength on the relationship between cognition and functional independence among Brazilian nonagenarians and centenarians.

**Methods:**

A cross‐sectional study was performed on 150 older adults aged > 90 years (141 nonagenarians and nine centenarians). A total of 105 participants (70%) were female. Cognitive function was assessed using the Mini‐Mental State Examination (MMSE), and functional independence was evaluated using the Katz Index score. The maximal isometric grip strength was measured using a handgrip dynamometer. A moderation analysis was performed to test whether handgrip strength moderated the association between cognition and functional independence, stratified by biological sex and adjusted for body weight.

**Results:**

Handgrip strength moderated the relationship between cognition and functional independence in both male and female older adults (*p* < 0.05). When handgrip strength was < 24.40 kg for males (42% of the study sample) and < 17.30 kg for female participants (60% of the study sample), the influence of cognitive function on functional independence was significant.

**Conclusion:**

Handgrip strength moderates the relationship between cognition and functional independence in Brazilian nonagenarians and centenarians. This attenuating effect of muscle strength on the impact of cognitive function on functional independence was observed in both weaker male and female individuals. Longitudinal studies are needed to validate our findings, as the cross‐sectional design limits the causal inferences.

## 1 Introduction

The faster growth of the nonagenarian and centenarian population is driven mainly by improved life expectancies among those aged 65 and older [1]. Overall, increased life expectancy has increased the likelihood of developing comorbidities, frailty, disability, dementia and advanced aging before death [2]. In contrast, nonagenarians and centenarians often exhibit medical histories with notably low incidence of age‐related disorders, including cardiovascular diseases, diabetes mellitus, Parkinson's disease and cancer [3], and most remain independent in activities of daily living (ADLs) until the 90s [4]. A heterogeneity in nonagenarians' and centenarians' physical and cognitive status seems to exist, depending on their socioeconomic status or cultural/historical background [5]. Moreover, aging‐related functional and cognitive impairment with subsequent substantial social and economic consequences has increased awareness of the relevance of research efforts focusing on enhancing the quality of life in the older population. In this context, the World Health Organization (WHO) has published the World Report on Aging and Health (S1), which identifies healthy aging as the interaction between an individual's physical and mental capacity (subdomains of intrinsic capacity) and the context of each individual's life (environment) (S2).

Several risk factors that predict the onset of cognitive impairment in the older population have been identified. The strongest included age, sex, educational level [6], genotype (family history of dementia or allele producing the e4 type of apolipoprotein E [APOE e4]) [7], parkinsonism [8], gait impairment [9] and hippocampal or medial temporal volume measures [10]. Regarding functional abilities, the highest strength of evidence for an increased risk of functional decline were cognitive impairment, depression, comorbidity burden, reduced muscle function (i.e., muscle strength and muscle mass), lower extremity mobility limitations, increased and decreased body mass index, loneliness, low level of physical activity, alcohol consumption, smoking, sensory impairments and poor self‐perceived health [11]. Although the causal relationship between cognitive impairment and functional decline appears bidirectional [12, 13], cognitive function significantly determines functional status in older adults [14]. Cognitive impairment has been associated with functional dependence on particular basic (e.g., bathing and toileting) and instrumental (e.g., using the telephone and taking medications) ADLs. Moreover, the highest level of cognitive impairment implies reduced performance in carrying out activities, with a more significant impact on basic ADLs [15]. Cognitive decline is a strong predictor of incident disability in specific ADL tasks among community‐dwelling older adults [16]. Furthermore, Barberger‐Gateau et al. observed that the risk of cognitive impairment (i.e., the incidence of dementia) over 1 year was higher in the presence of functional dependence evaluated by four instrumental ADLs in the older population [17]. Nevertheless, no studies have investigated possible third factors, such as muscle strength, in the associations between cognitive function and functional status in the oldest old (i.e., nonagenarians and centenarians).

Muscle function (e.g., strength and power) and mass decline with age. Aging‐related loss of muscle function is associated with significant impairment of functional status, promotion of muscle weakness and reduced ability to perform ADLs [18, 14]. Handgrip strength is the most widely used measure of muscle strength due to ease of assessment, low cost and simplicity and is considered a reliable ‘proxy’ of overall muscle strength for clinical and epidemiological research [19]. Multiple studies have determined the protective role of handgrip strength against ADL dependence in older adults [20, 21], and reduced handgrip force correlates with falls [22] and all‐cause mortality (S3, S4) [23]. Recent evidence has demonstrated the mediating role of functional independence in the association between handgrip strength and cognitive function in the older population [24].

Previous evidence demonstrated a positive association between cognitive function and functional abilities in older adults [12, 13]. Handgrip strength is considered an indicator of general vitality, and previous research has observed longitudinal associations between grip strength and cognitive [25] and functional abilities [26]. However, possible third factors (e.g., handgrip strength) that influence the association between both clinical characteristics remain unknown in the oldest age group.

Thus, we aimed to determine whether handgrip strength moderates the adverse effect of cognitive decline on the ability to perform ADLs in Brazilian adults over 90 years, stratified by biological sex. As muscle function seems to play a key role on the ability to perform ADLs, we hypothesized that handgrip strength would attenuate the relationship between cognition and functional independence in both males and females.

## 2 Methods

### 2.1 Design

This was a cross‐sectional study with nonprobabilistic sampling based on accessibility. Approval was obtained from the Research Ethical Committee of the University (CAAE: 79748517.5.0000.5347), the Research Ethical Committee of State Health Department (CAAE: 79748517.5.3002.5312) and the Research Ethical Committee of Municipal Health Department (CAAE: 79748517.5.3001.5338). All the participants signed a free and informed consent form.

### 2.2 Participants

The study sample included individuals aged ≥ 90 years residing in Porto Alegre, Rio Grande do Sul, Brazil. For sample size calculation, the odds ratio (OR) from ADL variable in the study by Dupre et al. [1] was selected. The authors used an OR of 2.49 with 0.95 power (Z‐tests Family). Additionally, since our study focuses on the health aspects of long‐lived older individuals, using the Dupre et al. [1] study for sample size estimation ensures that our sample calculation is based on a similar population. Finally, the sample size was calculated using GPOWER version 3.1.9.7 and was estimated to be 100 participants. The inclusion criteria were cognitive function ≥ 19 points on the Mini‐Mental State Examination (MMSE) and the ability to walk with or without assistance. Those who were unable to walk, relied on wheelchairs, had Parkinson's disease, severe sensory limitations or had amputations of the lower or upper limbs were excluded. The clinical and demographic characteristics of the study participants are presented in Table 1.

**TABLE 1:** Clinical and demographic characteristics of older individuals.

Variables	All (*n* = 150)	Males (*n* = 45)	Females (*n* = 105)
**Demographic data**			
Age, years	93.63 (3.34)	92.82 (2.83)	93.98 (3.49)
Body weight, kg	63.49 (13.20)	72.91 (12.16)	59.45 (11.49)*
Height, m	1.55 (0.10)	1.65 (0.07)	1.50 (0.06)*
BMI, kg/m^2^	26.39 (4.58)	26.68 (4.27)	26.27 (4.72)
**Study outcomes**			
MMSE, points Katz Index, points Handgrip strength, kg	25.67 (3.32)	26.44 (2.92)	25.34 (3.44)
	5.23 (1.14)	5.36 (1.05)	5.18 (1.18)
	18.17 (7.20)	25.51 (6.67)	15.02 (4.69)*

*Note:* Data are presented as the mean (SD) unless otherwise indicated.

Abbreviations: BMI, body mass index; MMSE, Mini‐Mental State Examination.

* Significant differences between male and female participants.

Participants were recruited for accessibility to Basic Health Units and public and private long‐term care institutions for older adults, senior living groups and religious institutions. Initial assessments of the participants were conducted via telephone. Following prior scheduling, assessments were conducted at home or in long‐term care institutions for older adults.

### 2.3 Measurements

Cognition was assessed using the MMSE (S5) validated for the Brazilian population (S6), which is composed of 11 open questions grouped into six categories: temporal and spatial orientation, processing, attention, calculation, evocation, language and constructive ability. The MMSE score is 0–30 points, with a score of ≤ 23 points indicatives of likely cognitive impairment (S7). Functional independence was evaluated using the Katz Index of Independence in ADLs (S8). This index ranks the adequacy of performance on the six functions of bathing, dressing, toileting, transferring, continence and feeding. A total score of 6 points indicated full physical function, 4 indicated moderate impairment and 2 or less indicated severe functional decline. The Katz Index was previously validated in the older population (S9, S10). Finally, handgrip strength was measured using a Hand Hydraulic Dynamometer (Saehan SH 5001) following the recommendations of the *American Society of Hand Therapists* (S11). The validity and reliability of the Saehan dynamometer have been confirmed previously (S12). The participants squeezed gradually and continuously for at least 2–3 s, performing the test with the right and left hand in turn three times. The maximum score in kg for each hand was recorded, and the mean score of the left and right hands was used in the analyses.

### 2.4 Statistical Analysis

Data are expressed as means and standard deviations (SD) for continuous variables and as numbers and percentages for categorical variables. After assessing the normal distribution of the variables, an independent *t*‐test was used to examine significant differences between male and female participants at baseline.

The moderating effect of upper limb muscle strength assessed by handgrip strength (moderator, M) on the relationship between cognitive (independent variable, X) and functional independence measured by the Katz Index (dependent variable, Y) was analysed using Andrew Hayes' PROCESS macro (S13). This relationship applies ordinary least squares regression analysis to predict continuous variables (handgrip strength and functional independence). A negative moderating effect indicates that the moderating variable (handgrip strength) attenuates the strength of the relationship between the independent (cognition) and dependent (functional independence) variables. Johnson‐Neyman plots show interactions between a constant exposure and modifier without using categorical modifier values by graphing the modifier values (x‐axis) against the beta for the exposure and outcome association (y‐axis). Thus, the Johnson‐Neyman technique identified areas of significance (*p* < 0.05) along the continuum of moderator values (handgrip strength), indicating that cognitive function significantly predicted functional independence. The analysis was adjusted for body weight. The statistical significance of the model was evaluated using bias‐corrected bootstrapping (*n* = 5000) and 95% confidence intervals (CI). The significance level was set at *p* < 0.05. SPSS statistical software version 27.0 for Windows (IBM Corp., Armonk, NY, USA) was used for statistical analysis.

## 3 Results

There were 150 older adults, with a mean (SD) age of 93.63 (3.34) years (range, 90–105 years), nine of whom (6%) were centenarians. At baseline, significant differences were observed in height, body weight and handgrip strength between male and female participants (*p* < 0.05).

### 3.1 Male Participants

**FIGURE 1:**
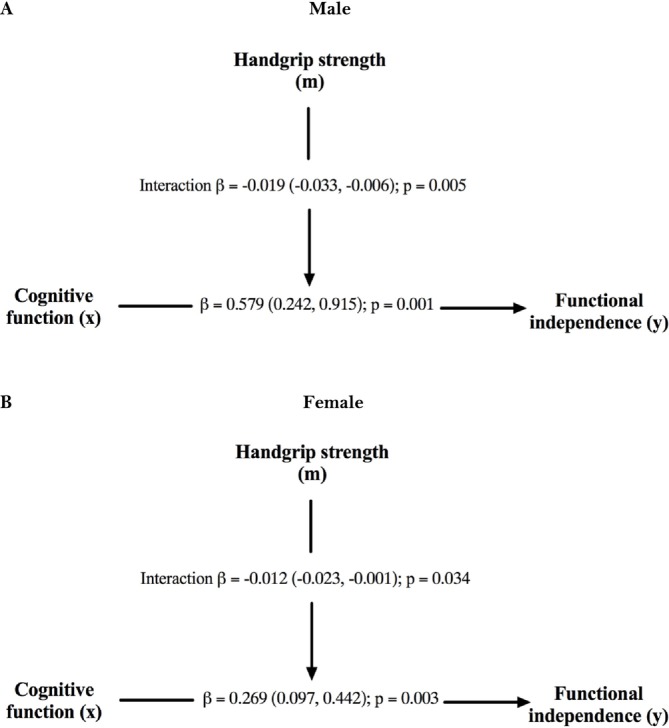
Moderation analyses of the relationship between cognitive function and functional independence, moderated by handgrip strength, in the oldest old. **Panel A** illustrated the findings for male participants, while **Panel B** shows the results for female participants. The moderation effect is represented by **path m** (handgrip strength), **path x** (cognitive function), and **path y** (functional independence). Beta coefficients (ß) are expressed as unstandardized coefficients with 95% confidence intervals. All analyses are adjusted by body weight.

**FIGURE 2:**
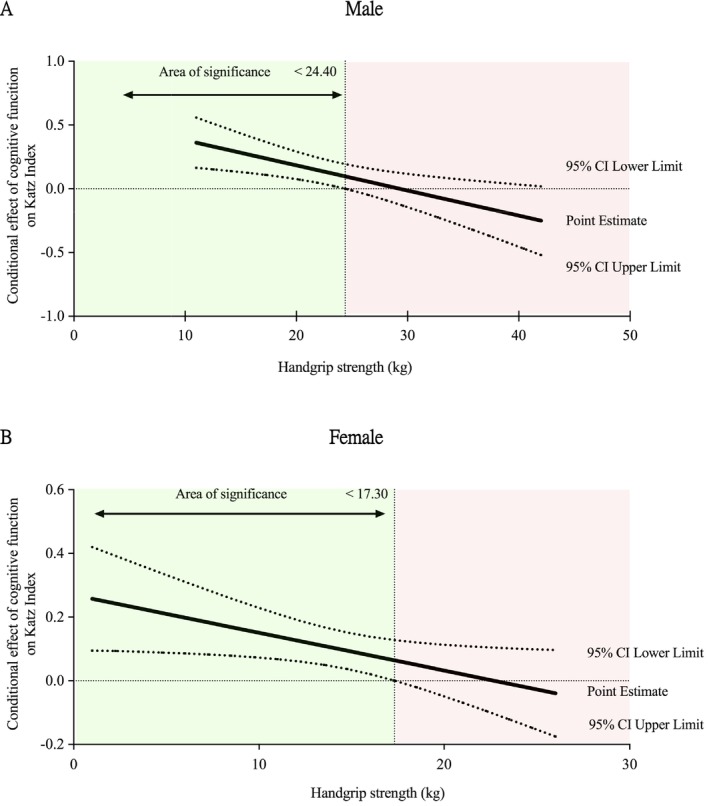
Moderation analyses using the Johnson‐Neyman technique in the oldest old. **Panel A** displays results for male participants, while **Panel B** presents findings for female participants. The Johnson‐Neyman technique identified areas of significance (*p* < 0.05) along the continuum of moderator values (handgrip strength), shown in green, indicating where cognitive function significantly predicts functional independence. Areas of nonsignificance are shown in red. Data are reported as regression point estimates (beta [ß] unstandardized) with 95% confidence intervals.

Figure 1A depicts our ordinary least squares regression moderation analysis for the oldest male participant, which showed a relationship between cognitive status (Path x) and functional independence (Path y). This path, known as a direct effect (*ß* = 0.579 [0.242, 0.915]; *p* = 0.001), was moderated by upper limb muscle strength as evaluated by handgrip strength. Therefore, the influence of cognition on the Katz Index was moderated by handgrip strength (interaction *ß =* −0.019 [−0.033, −0.006]; *p* = 0.005). We applied the Johnson‐Neyman statistical approach to clarify a possible estimate point where the moderator variable had a moderating effect (Figure 2A). The slope shows the continuum of the moderator (mean handgrip strength, represented as m) and area of significance. When handgrip strength was < 24.40 kg, the effect of cognitive status on functional independence was significant for male participants (42% in our study sample, *p* < 0.05). Above this value, no moderating effect of handgrip strength was observed between cognition and functional independence, and handgrip strength did not attenuate the relationship between independent and dependent variables.

### 3.2 Female Participants

Figure 1B shows the ordinary least squares regression moderation model for older women. A relationship was observed between the cognitive function (Path X) and functional status (Path Y). This association was established as a direct effect (ß = 0.269 [0.097, 0.442]; *p* = 0.003) moderated by handgrip strength. Therefore, the impact of cognition on functional independence was moderated by upper limb muscle strength (interaction *=* −0.012 [−0.023, −0.001]; *p* = 0.034). We obtained an estimate point to consider the moderating effect of the handgrip strength using the Johnson‐Neyman statistical method (Figure 2B). The slope represents the continuum of the moderator variable (i.e., handgrip strength) and the area of significance. In our study, the influence of cognition on functional status was significant when handgrip strength was below 17.30 kg in female participants (60% of the study sample, *p* < 0.05). No moderating effect of handgrip strength was observed beyond this value; thus, handgrip strength did not attenuate the association between cognitive function and functional independence.

## 4 Discussion

The primary finding of this observational, analytic and transversal study is that handgrip strength diminishes the effect of cognition, assessed using the MMSE test, on functional independence, evaluated using the Katz Index, in the oldest Brazilian population (i.e., nonagenarians and centenarians). The greater handgrip strength, the less significant the influence of cognitive function on functional independence, implying that cognition may have less impact on functional status when handgrip strength values are above 24.4 kg for male and 17.30 for female participants.

However, the causal association between cognition and functional status remains unclear. Cognitive impairment appears to influence the development of functional decline and *vice versa*, but the common mechanisms underlying both deterioration processes are still not well understood. Previous evidence has shown that older people with cognitive impairment are statistically more likely to be functionally dependent when measured using the ADL [15]. Given that impaired cognitive function is considered a potential indicator of functional dependence, identifying and understanding medical conditions and specific factors closely linked to functional disability could aid in assessing service requirements, enhancing the quality of life of older adults and alleviating the strain on caregivers and society. Our results reveal that muscle strength could be an essential therapeutic target for modifying the relationship between cognition and functional independence in older adults.

Muscle strength is an independent predictor of cognition and functional status in older adults [27, 28], which are components of the intrinsic capacity construct proposed by WHO. Handgrip strength has been recognized as an indicator of long‐term cognitive decline (i.e., 10 years) among community‐dwelling older adults [29]. Additionally, older adults with weak handgrip strength had an increased risk of adverse events in most intrinsic capacity domains (especially in cognition and locomotion domains, including falls and mobility limitations) and hospitalization [30]. For example, Ishizaki et al. determined that older adults with reduced handgrip strength had difficulty performing many ADLs, such as preparing meals, shopping for groceries and doing housework [31]. Previous studies have examined the effect of handgrip strength as a moderator variable between older individuals' demographic and clinical characteristics, such as age and sensory impairment [32] and excess adiposity and functional dependence [33], highlighting handgrip strength as a key component that could modify the associations between those variables. However, no previous studies have investigated the moderator role of muscle strength (i.e., handgrip strength) between cognitive function and functional independence in the oldest old (i.e., nonagenarians and centenarians).

Our study found a positive association between cognition and functional status. Additionally, handgrip strength moderates the relationship between cognition and functional independence in both male and female older adults. A novel finding of our study was the attenuating effect of handgrip strength on the association between cognitive function and functional independence in the weaker older individuals. These findings could be explained by several reasons and have important clinical implications. Muscle strength preservation may contribute to maintain functional independence in ADLs, even if the cognitive function is declining. A plausible mechanism explaining the association mentioned above could be that intrinsic muscle capacity preservation plays an essential role in promoting neuromuscular adaptations linked to increased functional performance in older adults [34]. Age‐related changes in the motor unit of the neuromuscular system and the neural inputs profoundly affect motor and cognitive function, especially in very old (older than 80 years) adults [35, 14]. Moreover, older adults with increased muscle strength seem to rely less on their cognitive abilities to maintain functional independence. In contrast, older individuals with low muscle strength may be compelled to depend more heavily on their cognitive performance to devise strategies that facilitate the maintenance of functional independence on ADLs [14]. Our results revealed that muscle strength significantly moderates the relationship between cognition and functional independence when an individual's handgrip strength is below 24.40 kg for males. For female participants, this moderating effect of handgrip strength was observed when the participant's upper limb muscle strength was below 17.30 kg. However, no moderating effect of handgrip strength was observed beyond these values in male and female participants. Thus, handgrip strength seems to reduce the impact of cognitive function on functional independence in both weaker males and females, showing that older individuals with greater muscle strength may depend less on their cognition to remain functional. This relationship suggests that in older adults with greater muscle strength, cognitive function is less critical for functional independence, whereas in those older individuals with low handgrip strength, cognition plays a much important role. Older individuals with increased handgrip strength may possess a more robust neuromuscular system, allowing them to perform ADLs with less reliance on executive function and other cognitive domains. Improved neuromuscular function and enhanced motor performance may reduce the cognitive load needed to maintain functional independence in the oldest old. Conversely, older adults with lower muscle strength are more dependent of cognitive resources (e.g., attention, planning and executive function) to compensate for their physical limitations. This is consistent with the Compensation‐Related Utilization of Neural Circuits Hypothesis (CRUNCH), suggesting that greater cognitive effort is required when physical systems are compromised (S15).

Several normative handgrip strength values for older adults from populations with different nationalities and ethnicities have been published in the last few years (S4) [21, 30, 36, 37, 38, 39]. Similarly, the European Working Group of Sarcopenia in Older People (EWGSOP) established sarcopenia cut‐off points for grip strength at 27 kg for males and 16 kg for older adults [40]. These cut‐off points were established from the normative data for grip strength across the life course obtained from 12 British studies (S14). Considering these data, we observed that the moderating strength in the relationship between cognition and functional status was significantly below the cut‐off points for male participants (i.e., 24.40 kg) and above (i.e., 17.30 kg) for female individuals in our study. Although previous studies have indicated normative values considering handgrip strength and the association with adverse events (i.e., mobility impairment, falls, institutionalization and mortality), our study extends this analysis and determines how handgrip strength could moderate the relationship between two essential clinical outcomes, cognition and functional independence. Physical exercise is an effective therapeutic strategy that contributes to muscle function improvement or maintenance and may preserve functional ability in ADLs in older adults, including those with cognitive impairment and dementia (S16). However, future studies are required to understand how exercise can induce changes in the moderating role of muscle strength in the relationship between cognition and functional independence in older adults.

This study has several limitations. First, owing to the cross‐sectional nature of the study, the potential to determine causal relationships was limited. Functional independence was evaluated using the Katz Index score, a subjective self‐report scale that includes only six ADLs (i.e., bathing, dressing, toileting, transferring, continence and feeding). Moreover, several physical impairments in this population (i.e., very old older adults) may not be fully captured using the Katz Index. Although nonagenarians and centenarians who can perform physical evaluations are not easily assessed, we could complete an objective muscle function assessment (i.e., handgrip strength) and cognitive evaluation using all of them. However, future studies should include more direct measures of physical function (e.g., physical performance tests such as gait speed, Timed Up and Go, Short Physical Performance Battery or Chair Stand Test) to assess the relationship between cognition and physical function and examine the moderating role of other factors (i.e., muscle strength).

Additionally, older adults with severe cognitive impairment (MMSE score < 19 points) were excluded from the study, affecting the relevance of the moderation analysis. Recruitment was performed in a single area of Brazil (Porto Alegre), and the small number of male individuals included (*n* = 45) reduced the generalizability of our findings. Finally, although we adjusted for body weight in our moderation analyses, other potential confounders may also have existed and biased the results, such as educational level and socioeconomic status (not registered in the data collection).

Nevertheless, our study has many strengths that need to be highlighted. We focused on the vulnerable older population (i.e., nonagenarians and centenarians). Moreover, our findings reinforced the role of muscle strength as a key biomarker in older adults, moderating the association between cognition and functional independence. Handgrip strength has been included as the core domain of sarcopenia [40] and the intrinsic capacity framework (S17), and previous evidence has emphasized the association with multiple adverse events, including mortality in this population [23]. Lastly, handgrip strength is an objective evaluation usually used for assessing the overall ‘proxy’ of muscle strength; therefore, the test is feasible to include in clinical practice.

In conclusion, our study shows that handgrip strength, a general indicator of muscle strength, moderates the relationship between cognition and functional independence in the oldest old Brazilian population (i.e., nonagenarians and centenarians). This attenuating effect of handgrip strength was observed in both weaker males and female individuals. Longitudinal and interventional studies are needed to validate our findings, as the cross‐sectional design limits the causal inferences.

## Ethics Statement

Approval was obtained from the Research Ethical Committee of the University (CAAE: 79748517.5.0000.5347), the Research Ethical Committee of State Health Department (CAAE: 79748517.5.3002.5312) and the Research Ethical Committee of Municipal Health Department (CAAE: 79748517.5.3001.5338). All the participants signed a free and informed consent form. The study followed the principles of the Declaration of Helsinki, and all the authors certify that they comply with the ethical guidelines for publishing in the Journal of Cachexia, Sarcopenia and Muscle: update 2019 [41].

## Conflicts of Interest

The authors declare no conflicts of interest.

## Supporting information


**Data S1.** Supporting Information.
