# Supplementary material for: Moderation Effect of Handgrip Strength on Cognition and Functional Independence Associations in Adults Over 90 Years
Source: J Cachexia Sarcopenia Muscle. 2025 Jun 4;16(3):e13838. doi: 10.1002/jcsm.13838 (PMC12134779; doi:10.1002/jcsm.13838)
Supplement: Supplementary file 1 — Data S1. Supporting Information. [file JCSM-16-e13838-s001.docx]

**SUPPLEMENTARY REFERENCES**

S1. World Health Organization. World report on ageing and health. 2015; Geneva: World Health Organization.

S2. Beard JR, Officer A, de Carvalho IA, et al. The world report on ageing and health: a policy framework for healthy ageing. *Lancet*. 2016;387(10033):2145-2154. doi:10.1016/S0140-6736(15)00516-4

S3. López-Bueno R, Andersen LL, Koyanagi A, et al. Thresholds of handgrip strength for all-cause, cancer, and cardiovascular mortality: a systematic review with dose-response meta-analysis. *Ageing Res Rev*. 2022;82:101778. doi:10.1016/j.arr.2022.101778

S4. Andersen LL, López-Bueno R, Núñez-Cortés R, Cadore EL, Polo-López A, Calatayud J. Association of muscle strength with all-cause mortality in the oldest old: prospective cohort study from 28 countries. *J Cachexia Sarcopenia Muscle*. 2024. doi:10.1002/jcsm.13619

S5. Folstein MF, Folstein SE, McHugh PR. “Mini-mental state”. A practical method for grading the cognitive state of patients for the clinician. *J Psychiatr Res*. 1975;12(3):189-198. doi:10.1016/0022-3956(75)90026-6

S6. Bertolucci PH, Brucki SM, Campacci SR, Juliano Y. The Mini-Mental State Examination in a general population: impact of educational status. *Arq Neuropsiquiatr*. 1994;52(1):1-7.

S7. Folstein M, Anthony JC, Parhad I, Duffy B, Gruenberg EM. The meaning of cognitive impairment in the elderly. *J Am Geriatr Soc*. 1985;33(4):228-235. doi:10.1111/j.1532-5415.1985.tb07109.x

S8. Wallace M, Shelkey M. Katz index of independence in activities of daily living. *Urol Nurs*. 2007;27(1):93-94.

S9. Arik G, Varan HD, Yavuz BB, et al. Validation of Katz index of independence in activities of daily living in Turkish older adults. *Arch Gerontol Geriatr.* 2015;61(3):344-350. doi:10.1016/j.archger.2015.08.019

S10. Shelkey M, Wallace M. Katz Index of Independence in Activities of Daily Living (ADL). *Director*. 2000;8(2):72-73.

S11. Fess EE. Grip strength In: Casanova JS, editor. *Clinical Assessment Recommendations*; 2.

S12. Reis MM, Arantes PMM. Assessment of hand grip strength- validity and reliability of the saehan dynamometer. *Fisioter Pesqui.* 2011;18:176-181. doi:https://doi.org/10.1590/S1809-29502011000200013

S13. Hayes AF. Partial, conditional, and moderated moderated mediation: quantification, inference, and interpretation. *Commun Monogr*. 2018;85(1):4-40. doi:10.1080/03637751.2017.1352100

S14. Dodds RM, Syddall HE, Cooper R, et al. Grip strength across the life course: normative data from twelve British studies. *PLoS One*. 2014;9(12):e113637. doi:10.1371/journal.pone.0113637

S15. Van Ruitenbeek P, Santos Monteiro T, Chalavi S, et al. Interactions between the aging brain and motor task complexity across the lifespan: balancing brain activity resource demand and supply. *Cereb Cortex*. 2023;33(10):6420-6434. doi:10.1093/cercor/bhac514

S16. Izquierdo M, Duque G, Morley JE. Physical activity guidelines for older people: knowledge gaps and future directions. *Lancet Healthy Longev*. 2021;2(6):e380-e383. doi:10.1016/S2666-7568(21)00079-9

S17. Cesari M, Araujo de Carvalho I, Amuthavalli Thiyagarajan J, et al. Evidence for the domains supporting the construct of intrinsic capacity. *J Gerontol A Biol Sci Med Sci*. 2018;73(12):1653-1660. doi:10.1093/gerona/gly011
